# Expression of protease activated receptor-2 is reduced in renal cell carcinoma biopsies and cell lines

**DOI:** 10.1371/journal.pone.0248983

**Published:** 2021-03-25

**Authors:** Christudas Morais, Retnagowri Rajandram, Jade S. Blakeney, Abishek Iyer, Jacky Y. Suen, David W. Johnson, Glenda C. Gobe, David P. Fairlie, David A. Vesey

**Affiliations:** 1 Centre for Kidney Disease Research, The University of Queensland, Translational Research Institute, Brisbane, Australia; 2 Department of Urology, Princess Alexandra Hospital, Brisbane, Australia; 3 Department of Surgery, Faculty of Medicine, University of Malaya, Kuala Lumpur, Wilayah Persekutuan, Malaysia; 4 Centre for Inflammation and Disease Research, Institute for Molecular Bioscience, The University of Queensland, Brisbane, QLD, Australia; 5 Department of Nephrology, Princess Alexandra Hospital, Brisbane, Australia; University of Alabama at Birmingham, UNITED STATES

## Abstract

Expression of the protease sensing receptor, protease activated receptor-2 (PAR2), is elevated in a variety of cancers and has been promoted as a potential therapeutic target. With the development of potent antagonists for this receptor, we hypothesised that they could be used to treat renal cell carcinoma (RCC). The expression of PAR2 was, therefore, examined in human RCC tissues and selected RCC cell lines. Histologically confirmed cases of RCC, together with paired non-involved kidney tissue, were used to produce a tissue microarray (TMA) and to extract total tissue RNA. Immunohistochemistry and qPCR were then used to assess PAR2 expression. In culture, RCC cell lines versus primary human kidney tubular epithelial cells (HTEC) were used to assess PAR2 expression by qPCR, immunocytochemistry and an intracellular calcium mobilization assay. The TMA revealed an 85% decrease in PAR2 expression in tumour tissue compared with normal kidney tissue. Likewise, qPCR showed a striking reduction in PAR2 mRNA in RCC compared with normal kidney. All RCC cell lines showed lower levels of PAR2 expression than HTEC. In conclusion, we found that PAR2 was reduced in RCC compared with normal kidney and is unlikely to be a target of interest in the treatment of this type of cancer.

## Introduction

Renal cell carcinoma (RCC) accounts for about 3% of adult malignancies [1, 2]. However, its incidence has reportedly been increasing at 2–3% per decade [1, 2]. Together with the highly drug-resistant nature of this cancer, and the fact that diagnosis usually occurs at advanced stages due to lack of early warning signs, understanding the molecular pathogenesis of RCC and refining diagnostic and treatment options are of great importance [3]. The modification of extracellular matrix components by extracellular proteases plays a critical role in tumour progression, invasion and metastasis [4–13]. The release of proteases and their activation in the extracellular environment often occur following tissue injury, inflammation, infection and malignancy and lead to tissue remodelling and activation of matrix bound growth factors [10, 14]. This, in turn, leads to modification of the cellular microenvironment, which enhances cancer progression. In addition, certain serine proteases can activate cell surface receptors called protease-activated receptors (PARs), which can enhance tumour growth [15, 16].

PARs constitute a unique sub-family of G-protein coupled receptors (GPCR) that function as extracellular protease sensors [15, 17]. Their activation involves proteolytic cleavage of their extracellular domain and binding of the newly exposed “tethered ligand” to a membrane-spanning segment of the receptor. This activation leads to intracellular signalling, which allows the cell to respond appropriately to threats posed by locally released proteases. Responses of cells to proteases that are mediated by PARs have consistently been reported to involve pro-inflammatory cytokine production, cell proliferation and cell migration [18, 19]. The second member of this family, PAR2, is activated by trypsin-like proteases including tryptase and trypsin [17, 20]. PAR2 expression is upregulated in a wide variety of cancers of the skin, prostate, breast, stomach, pancreas, ovaries, colon and kidney [16, 21–29]. Multiple *in vitro* studies of various cancer cell lines have demonstrated that PAR2 activation leads to cell migration, cell proliferation and angiogenesis [30]. The combination of enhanced protease production and PAR2 expression could facilitate an autocrine feedback loop enhancing tumour growth. Thus, blocking the activation of PAR2 in the setting of malignancy may be an effective cancer treatment option [31, 32].

Within the kidney, the highest level of PAR2 expression is found in the proximal tubular epithelium, the source of cells that form RCC when transformed. In addition, two recent publications have demonstrated that PAR2 expression is elevated in RCC [26, 27]. Thus, we were encouraged to investigate whether increased PAR2 expression could enhance RCC growth and progression. If so, we planned to use highly potent and bioavailable PAR2 antagonists [33–35] to test the functional role of increased PAR2 expression in RCC cell lines and in a mouse xenograft model. In the present study, we examined PAR2 expression in our a bank of human kidney cancer tissues and cell lines as a forerunner to evaluating therapeutic effects of PAR2 antagonists.

## Results

### PAR2 expression in human RCC tissue

**Fig 1:**
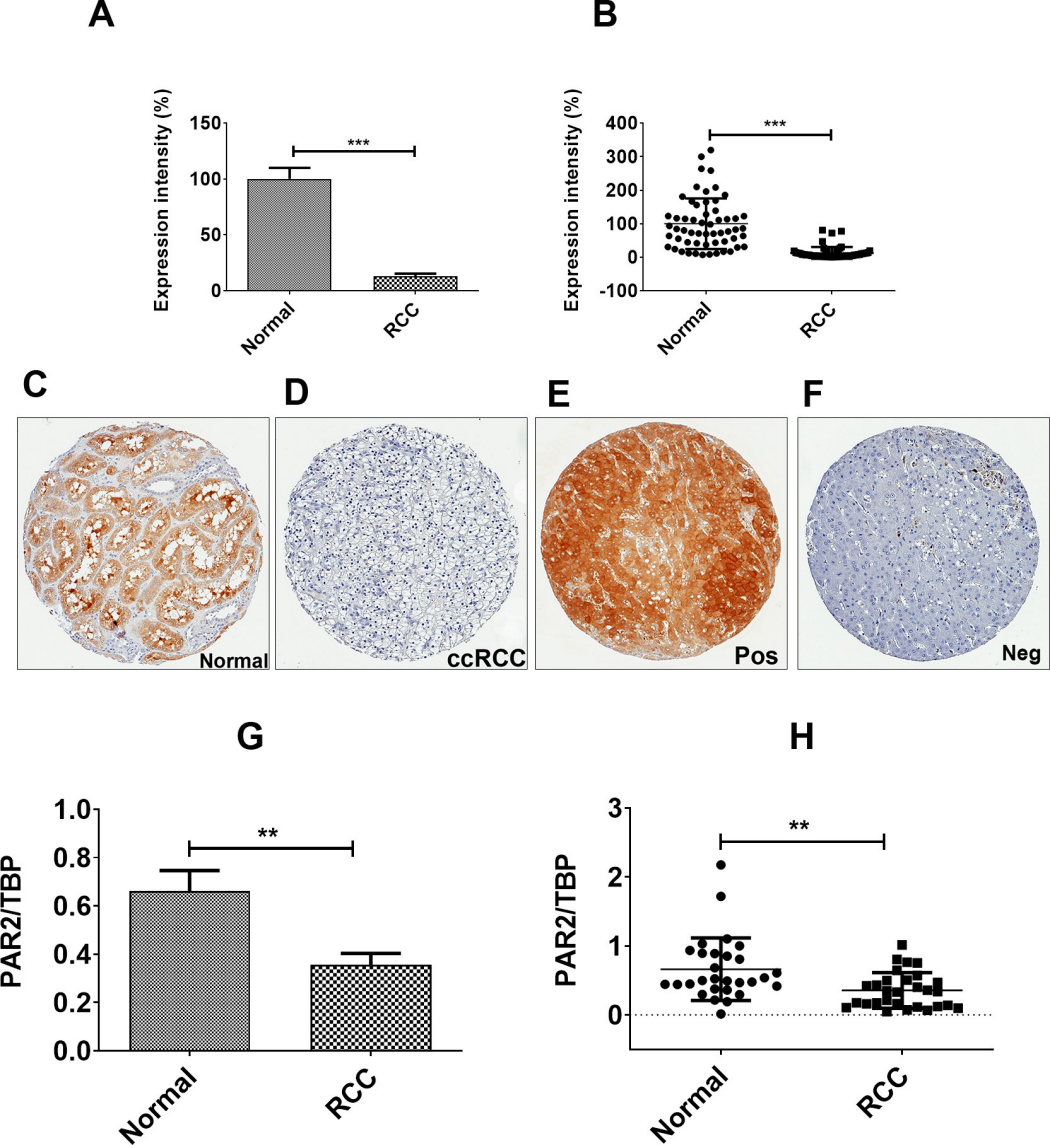
PAR2 expression in renal cell carcinoma. PAR2 protein was examined in human clear cell RCC tissue samples and paired normal kidney tissue by immunohistochemistry. Average expression intensity of the staining is compared in tumour tissue samples and paired normal tissue samples (A). Expression intensity for each tissue sample is shown as a dot plot (B). Examples of the PAR2 staining are demonstrated for normal kidney tissue (C), clear cell RCC tissue (ccRCC; D), positive control (Pos; liver; E), and negative control (Neg; liver; F). In negative control, the primary antibody was excluded. qPCR results show the average expression of PAR2 mRNA compared to the house keeping gene TBP (G). The mRNA expression of each sample is shown as a dot plot (H). In all examples, PAR2 was reduced in RCC compared with normal kidney.

Tissue microarrays (TMA) consisting of 57 clear cell RCC along with their paired normal kidney tissue were analysed. There was an estimated 85% reduction of PAR2 (p < 0.001) expression in all RCC samples compared with non-cancer kidney tissue controls (Fig 1A). Individual dot plots of the samples are presented in Fig 1B. Representative images of normal (Fig 1C), clear cell RCC (Fig 1D), and liver cores as positive control (Fig 1E) and negative controls (Fig 1F) are also shown. To verify these findings, mRNA expression of PAR2 was performed in 29 different clear cell RCC tissues along with matched normal samples. In line with the TMA results, there was an overall decrease in PAR2 in RCC samples (Fig 1G), with a degree of heterogeneity as shown in the dot plot (Fig 1H).

### PAR2 expression and Ca^2+^ mobilization in RCC cell lines

**Fig 2:**
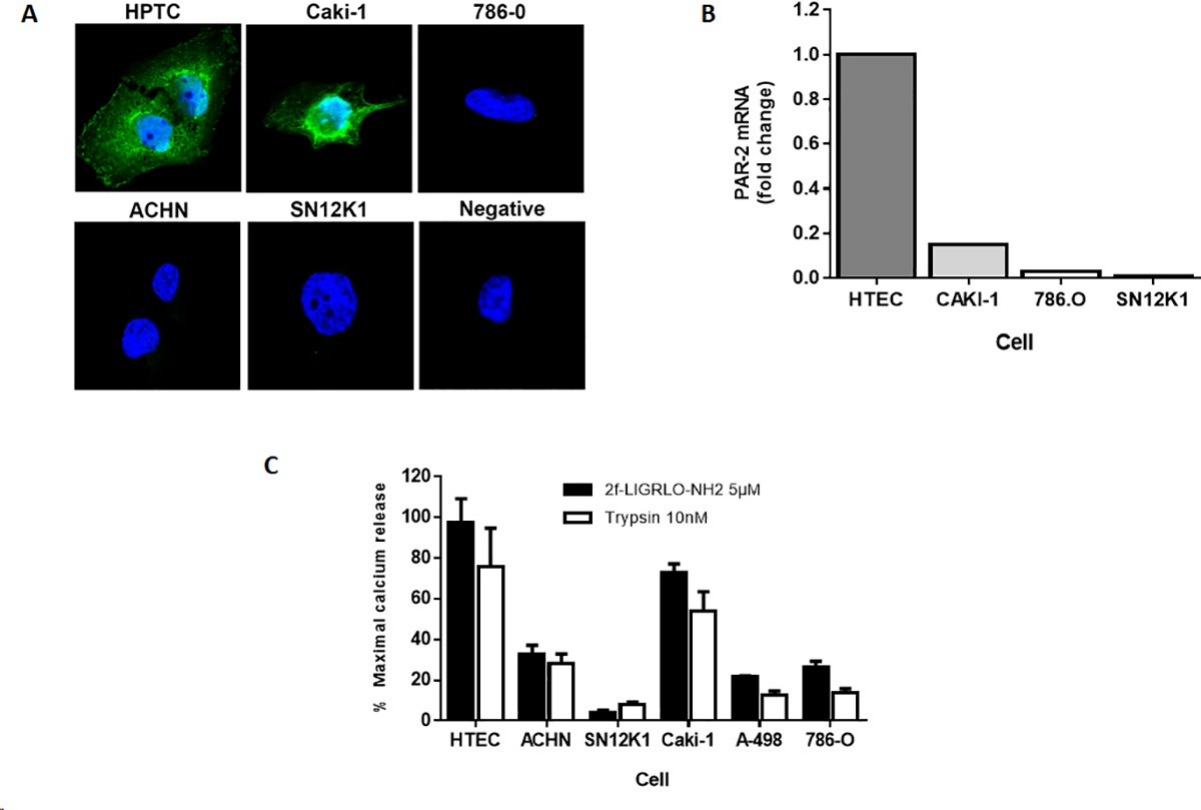
PAR2 expression in human RCC cell lines and primary kidney HTEC. PAR2 protein expression was examined in the cell lines by immunocytochemistry (A), qPCR. (B) and intracellular calcium mobilization (n = 3, mean ± SEM) (C). Confocal microscopy of PAR2 expression was significantly reduced in all cell lines compared with primary human tubular epithelial cells (HTEC), with Caki-1 having highest expression (2A). PAR2 mRNA (2B) was highest in HTEC with significantly reduced mRNA in all RCC cell lines. The PAR2 agonists 2F and trypsin induce significantly less intracellular Ca^2+^ mobilization in the five human cancer cell lines than in HTEC (2C).

The expression of PAR2 in five human RCC cell lines, ACHN, Caki-1, 786-O, A-498, SN12K1 and for comparison, primary human kidney tubular epithelial cells (HTEC), was measured by three different techniques; confocal microscopy, qPCR and a Ca^2+^ ratiometric assay using the fura-2 probe (Fig 2). In each of these assays, the HTEC expressed much higher levels of PAR2 than the RCC cell lines. By immunofluorescence and confocal microscopy, PAR2 was detectable in only the Caki-1 and HTEC cells and was prominent in the perinuclear region of these cells (Fig 2A). By qPCR, the Caki-1 cells expressed the highest level of PAR2 mRNA of the RCC cell lines, with the SN12K1 cells having least PAR2 (Fig 2B). Compared to HTEC, however, even Caki-1 cells had lower expression of PAR2 (Fig 2B). The PAR2 agonist 2-furoyl-LIGRLO-NH_2_ (2F) and trypsin induced variable but robust intracellular Ca^2+^ mobilisation in all cells. The highest response was from the HTEC (primary cells) and the lowest SN12K1 cells (Fig 2C).

## Discussion

Multiple *in vitro* studies suggest a role for PAR2 in cancer development, including induction of cell migration, proliferation, metastasis and angiogenesis [30, 36, 37]. These reports of enhanced expression of PAR2 in cancer pathogenesis have led to it being promoted as a drug target worthy of investigation [26, 27]. Potent, selective and bioavailable PAR2 antagonists have shown therapeutic efficacy in animal models of inflammatory bowel disease (IBD), arthritis and glomerulonephritis [18, 34, 35, 38]. This has raised the possibility that PAR2 antagonists might be useful in the treatment of cancers where PAR2 is overexpressed.

Publications by Sun *et al* and Zhang *et al* [26, 27] reported a significant increase in PAR2 expression in RCC tumour tissues. Sun *et al* [26] found that PAR2 was overexpressed in RCC cells in culture, and activation of PAR2 using agonist peptides enhanced the invasion and migration of RCC cells. Knockdown of PAR2 by siRNA modulated all effects of the PAR2 activating peptide. They reported that activation of the PI3K/AKT pathway was central to promoting the invasion and migration of RCC cells. Zhang *et al* [27] investigated the relationship between pro-angiogenic growth factors, PAR2 and RCC development. In their studies, RT-PCR and immunohistochemistry assays also showed that PAR2 expression was significantly increased in human RCC tissue compared with the adjacent non-neoplastic kidney tissue. PAR2 was functional in RCC as assessed using signalling through MAP kinases including ERK1/2 and JNK. They also found that PAR2 significantly upregulated expression of IL-6, IL-8, monocyte chemotactic protein-1 and growth-related oncogene, all considered pro-angiogenic factors that contribute to RCC progression. The PAR2 SAM-11 antibody had been used by Sun *et al* and Zhang *et al*. [26, 27], and by other groups demonstrating enhanced PAR2 expression in tumour tissue [22, 26, 27, 39–41]. For this reason, we planned to use the same antibody in our immunobiological study.

In contrast with publications by Sun *et al* and Zhang *et al* using RCC tissue, we found PAR2 expression to be significantly lower in RCC tissue compared with paired normal kidney tissue [26, 27]. Thus, we decided to augment our investigations of PAR2 expression using qPCR. We used a second set of paired normal kidney and RCC samples from our kidney cancer biobank and again the expression of PAR2 was found to be significantly lower in RCC tissue compared with the paired normal kidney. This confirmed our findings using IHC.

The discrepancy between our study and others was perplexing [26, 27]. As RCC are highly heterogeneous cancers, it is possible that PAR2 expression is different in different cohorts of kidney cancer patients and we did observe some variability among samples used in the present study. It is also possible that, because our expression assays were not designed to determine functional roles such as angiogenesis and invasiveness [26, 27], disparity in expression levels might have occurred. Nonetheless, when we examined PAR2 expression in five RCC cell lines, we found the same result of reduced PAR2 compared with primary HTEC. Additionally, variability of PAR2 expression amongst the RCC cell lines was observed. The Caki-1 cells had the highest level of PAR2 expression of the immortalised RCC cell lines examined and SN12K1 the lowest.

A limitation of this study is that it was descriptive in nature. Thus, a definitive conclusion about a role PAR2 in this cancer type cannot be made. Our initial aim was to test recently developed PAR2 antagonists, I-191 and GB88, together with a PAR2 expressing RCC cell line, in a mouse xenograft model RCC [42, 43]. As it was found that RCC cell lines express relatively low levels of PAR2 we did not peruse this further. However, by using a RCC cell line that artificially over express PAR2 within this experimental design it may be mechanistically be informative.

In summary, PAR2 expression was found to be significantly reduced in RCC tissues compared with normal kidney tissue, as revealed by IHC and qPCR. PAR2 expression was also reduced, compared to primary HTEC, in all the established RCC cell lines examined. PAR2 is thus unlikely to be a potential therapeutic target in this type of cancer.

## Materials and methods

### Ethics statement

The use of human kidney tissue for this study was reviewed and approved by the Princess Alexandra Hospital (PAH) Human Research Ethics Committee (HREC/12/QPAH/125 and HREC/16/QPAH/353). Patient consent was obtained before surgery and any tissue collection. For the purposes of this study, archived paraffin embedded tissue samples were used from a collection made between the years 1990 and 2011. Only limited de-identified clinical data was available for these samples.

### Cell culture

Human RCC cell lines ACHN, Caki-1, A498 and 786-O, were obtained from the American Type Culture Collection (ATCC), Rockville, MD, USA. Another human metastatic RCC cell line, SN12K1, was obtained from Professor D Nicol, formerly of the PAH, Brisbane, Australia, through his collaborations with Professor I.J. Fidler, University of Texas MD Anderson Cancer Center in Houston, Texas, USA. At the time of experimentation all cell lines were free of mycoplasma contamination. The cell lines were cultured in DMEM/F12 supplemented with 10% foetal bovine serum, 100 U/ml penicillin and 100 μg/ml streptomycin (Gibco, Invitrogen, CA, USA) at 37°C in an atmosphere of 95% air and 5% carbon dioxide. Primary cultures of morphologically normal human proximal tubular epithelial cells (HTEC) were established by us and propagated as previously described [19, 20, 44].

### Tissue microarray

Fifty-seven formalin-fixed paraffin-embedded (FFPE) archival kidney cancer and matched morphologically normal regions of the kidneys were used in this study. They were collected from patients who underwent nephrectomy for kidney cancer between 1990 and 2011 at the PAH, Brisbane, Australia. None of the patients received prior treatment before nephrectomy. Tumour grade and stage were determined by Fuhrman criteria and TNM classification, respectively, by a qualified Pathologist to ensure tumour regions were not contaminated by morphologically normal regions and vice versa. Tissue samples (0.6 mm in diameter) were punched from selected areas of the paraffin blocks with core punch needles (Beecher Instruments, Inc. Sun Prairie, WI, USA) and tissue microarrays (TMA) of were constructed using a Galileo TMA CK3000 Tissue Microarrayer (Fantoli, Milan, Italy).

### Immunohistochemistry of TMA

TMA were cut at 5 μm onto Superfrost Plus histology slides. A haematoxylin and eosin-stained slide was used to confirm the array spots by a pathologist blinded to positioning of samples to ensure that normal samples were not contaminated with cancer cells and vice versa. The slides were then immune-stained for PAR2 expression as per routine immunohistochemistry (IHC) procedure. In brief, the TMAs were de-paraffinized in xylene, rehydrated in successive 100%, 95% and 75% ethanols and washed in Tris-buffered saline (TBS). After unmasking antigens at 100°C in 10% sodium citrate buffer (pH 6.5) for 10 min, the endogenous peroxidase activity was quenched by incubation in 0.3% hydrogen peroxide (H_2_O_2_)/0.1% sodium azide in TBS for 10 min. The TMA were blocked in horse serum (1:100 in freshly prepared 0.1% bovine serum albumin in TBS for 60 min at room temperature) and were incubated at 4°C overnight with or without (negative control) the PAR2 antibody (Santa Cruz sc-13504), also used in [26, 27]. After washing in TBS (3 × 5 min each), the TMA were incubated with Horseradish peroxidase-conjugated secondary antibody (Thermofisher Scientific, MA USA) for 30 min at room temperature. The sections were washed in TBS, developed with 3,3′ diaminobenzidine for 2–3 min, lightly counterstained with haematoxylin, dehydrated in ethanols, cleared in xylene and mounted with coverslips using DePex mounting medium (Searle Diagnostics, High Wycombe, Bucks, UK). The TMA were batch-stained. Negative controls (omitting primary antibody) were used with each batch. Liver core samples were used as positive tissue controls. The IHC procedure was performed using a Bond-Max automated immunostainer (Vision BioSystems, Australia) in the Histology Unit, Queensland Institute for Medical Research, Brisbane, Australia. The kit used for IHC was a Bond Polymer Refine Detection kit (Vision Biosystems, Cat. # DS9800). Thus, the slides were stained in a constant environment, making comparisons in expression patterns among samples in the TMA as controlled as possible. Negative controls were also stained in a similar fashion except that the primary antibody was excluded.

### Analysis of TMA

Digital images of the entire array of spots were captured using the Aperio ScanScope XT Slide Scanner (Aperio Technologies, Vista, CA, USA) under 20 × objective magnification. A quantitative scoring of PAR2 expression of the whole spot was analysed using the positive pixel algorithm of Aperio ImageScope [45]. Based on the staining intensity of the tissue, the Aperio Positive Pixel Count v9 algorithm generates an automated positive pixel count (positive and negative pixels %). The intensity output was exported to an Excel spread sheet and the difference in expression intensity (%) between normal and tumour regions were calculated. GraphPad Prism 6 (GraphPad Software) was used to calculate significance and generate graphs. Graphs were generated to show the % expression change for tumour versus normal kidney. Since there were only two groups (normal and RCC), significance of differences was analysed with Student’s t-test. P<0.05 was considered significant. For quality assurance, all TMA spots were further evaluated visually by a pathologist to provide a semi-quantitative estimate of staining intensity.

### PAR2 mRNA expression studies

RCC and matched normal regions of kidneys were collected from 51 patients who underwent nephrectomy for kidney cancer between June 2013 and December 2014 at the PAH. Of these 29 where verified to be from clear cell renal carcinoma cases. None of the patients received prior treatment before nephrectomy. The samples were snap-frozen in liquid nitrogen and stored at -80 ^o^C until further use. RNA from tissue samples and the cell lines was isolated using RNeasy Fibrous Tissue Mini Kit and RNeasy mini kit, respectively, following the instructions of the supplier (Qiagen, Hilden, Germany). cDNA was synthesised using High Capacity cDNA Reverse Transcription Kit (Thermofisher Scientific, MA USA). Fully validated TaqMan Gene Expression Assay for PAR2 (Hs00608346_m1; Thermofisher Scientific, MA USA) was used with the SensiFAST™ Probe No-ROX Kit (Bioline, London, UK) and LightCycler 480 (Roche Applied Science, Penzberg, Germany) to determine relative gene expression by the comparative Ct method. The TATA box binding protein (TBP Hs00427620_m1) was used as internal control.

### Confocal microscopy

Cells were grown on glass cover slips overnight and washed in PBS, fixed in 1% paraformaldehyde for 15 min at room temperature, permeabilized in 0.1% Triton X-100 for 3 min, washed in TBS, and blocked with serum-free protein block (DakoCytomation, CA, USA; Catalogue number, XO909) at room temperature for 1 h. The PAR2 primary antibody (Santa Cruz sc-13504), was diluted (1:1000) in antibody diluent (DakoCytomation, CA, USA; Catalogue number, S0809), and the cells were incubated with the antibodies for 1 h at room temperature. After washing in PBS, the cells were labelled with FITC-conjugated secondary antibodies (Thermofisher Scientific, MA USA) for 30 min, washed in PBS and mounted in Vectashield mounting medium (Vector laboratories Inc. CA, USA; Catalogue number, H-1200). The cells were observed under a Nikon Eclipse E800 microscope (40 x objective).

### Intracellular calcium mobilization

Cells were grown to 80% confluence. After removal of the supernatant, cells were incubated in dye loading buffer (HBSS with 4 μM Fluo-3, 25 μL pluronic acid, 1% fetal bovine serum and 2.5 mM probenecid) for 1 h at 37°C. Cells were then washed twice with HBSS and transferred to a Polarstar spectrofluorimeter (BMG, Durham, NC, USA) for PAR2 agonist injection (2-furoyl-LIGRLO-NH_2_/2f-LIGRLO-NH_2_, manufactured in-house) and fluorescence measurements. The PAR2 agonist was added 10 s after reading commenced and fluorescence was measured in real time from the bottom of the plate using excitation at λ = 480 nm and emission at 520 nm. HBSS was prepared in-house. All other reagents and calcimycin (A23187) were purchased from Invitrogen (Carlsbad, CA, USA), the latter for measuring maximum fluorescence. Plates were purchased from DKSH (Zurich, Switzerland).

### Statistical analysis

All data was analysed by GraphPad Prism 8 (San Diego, CA, USA) and presented as the mean + SEM. Statistical significance (*p<0.05, **p<0.01, *** p < 0.001) was determined using Students t-test. All the cell culture experiments were performed in triplicate.

## Supporting information

S1 File (PDF)

S2 File (PDF)
