# Supplementary material for: Expression of protease activated receptor-2 is reduced in renal cell carcinoma biopsies and cell lines
Source: PLoS One. 2021 Mar 25;16(3):e0248983. doi: 10.1371/journal.pone.0248983 (PMC7993771; doi:10.1371/journal.pone.0248983)
Supplement: S1 File — (PDF) [file pone.0248983.s001.pdf]

Region	ID	Normal Total Intensity	Normal Total Intensity %	Region	Ip = Total Intensity of Positive	Tumor Total Intensity %
Normal	TMA1 A3 cc2	21180958	93.38	Tumour	628724	2.77
Normal	TMA1 A4 cc3	10672911	47.06	Tumour	10672911	47.06
Normal	TMA1 A5 cc1	27814844	122.63	Tumour	5331595	23.51
Normal	TMA1 A6 cc1	14435911	63.65	Tumour	499295	2.20
Normal	TMA1 A7 cc2	9818080	43.29	Tumour	2944872	12.98
Normal	TMA1 A8 cc2	9438350	41.61	Tumour	144272	0.64
Normal	TMA1 B1 cc2	41040724	180.94	Tumour	1525132	6.72
Normal	TMA1 B4 cc2	19163789	84.49	Tumour	6882682	30.34
Normal	TMA1 B6 cc2	17425527	76.83	Tumour	2441113	10.76
Normal	TMA1 C1 cc3	25162223	110.94	Tumour	958309	4.23
Normal	TMA1 C2 cc2	25712687	113.36	Tumour	1580551	6.97
Normal	TMA 1 C7 cc2	12539459	55.28	Tumour	5052466	22.28
Normal	TMA 1 D3 cc2	19492622	85.94	Tumour	3078980	13.57
Normal	TMA 1 D4 cc2	16428367	72.43	Tumour	1831136	8.07
Normal	TMA1 D7 cc2	10127973	44.65	Tumour	763677	3.37
Normal	TMA1 F1 cc3	2441721	10.77	Tumour	6054493	26.69
Normal	TMA1 F2 cc1	37336550	164.61	Tumour	439994	1.94
Normal	TMA1 F3 cc2	42093387	185.58	Tumour	1521085	6.71
Normal	TMA1 F7 cc3	25252063	111.33	Tumour	3051230	13.45
Normal	TMA1 F8 cc2	25949262	114.41	Tumour	304807	1.34
Normal	TMA1 G1 cc4	22269071	98.18	Tumour	984089	4.34
Normal	TMA1 G6 cc2	59848654	263.86	Tumour	6230708	27.47
Normal	TMA1 G7 cc2	18116808	79.87	Tumour	1768999	7.80
Normal	TMA1 H2 cc4	16585994	73.13	Tumour	1077059	4.75
Normal	TMA1 H3 cc2	28939805	127.59	Tumour	1237225	5.45
Normal	TMA1 H7 cc2	27862287	122.84	Tumour	1082705	4.77
Normal	TMA1 H8 cc2	15704969	69.24	Tumour	2.84E+06	12.51
Normal	TMA1 I1 cc2	35306287	155.66	Tumour	359450	1.58
Normal	TMA1 I2 cc3	7018977	30.95	Tumour	4829440	21.29
Normal	TMA1 I3 cc2	26406806	116.42	Tumour	227370	1.00
Normal	TMA1 I5 cc2	5525384	24.36	Tumour	318291	1.40
Normal	TMA1 I8 cc2	8391289	37.00	Tumour	718549	3.17
Normal	TMA1 J1 cc2	3785111	16.69	Tumour	4592746	20.25
Normal	TMA1 J3 cc2	16057307	70.79	Tumour	1105637	4.87
Normal	TMA1 J7 cc2	12747253	56.20	Tumour	1899891	8.38
Normal	TMA 1 J8 cc2	23252538	102.52	Tumour	4418223	19.48
Normal	TMA 2 A3 cc1	2739755	12.08	Tumour	997250	4.40
Normal	TMA2 A8 cc1	31468074	138.74	Tumour	2737604	12.07
Normal	TMA2 B1 cc1	37831246	166.79	Tumour	2663204	11.74
Normal	TMA2 B6 cc4	15910081	70.15	Tumour	182090	0.80
Normal	TMA2 B8 cc2	14411143	63.54	Tumour	1799242	7.93
Normal	TMA2 C1 cc2	3815833	16.82	Tumour	607651	2.68
Normal	TMA2 C2 cc2	68034855	299.96	Tumour	4303315	18.97
Normal	TMA2 C7 cc3	1689598	7.45	Tumour	516074	2.28
Normal	TMA2 C8 cc2	7089810	31.26	Tumour	1171052	5.16
Normal	TMA2 D1 cc3	3.83E+07	168.82	Tumour	2422179	10.68

Normal	TMA2 D3 cc2	1924901	8.49	Tumour	5.63E+05	2.48
Normal	TMA2 D4 cc3	44512238	196.25	Tumour	2.48E+06	10.92
Normal	TMA2 D6 cc2	4.72E+07	208.15	Tumour	1595444	7.03
Normal	TMA2 F2 cc4	3889723	17.15	Tumour	4.50E+05	1.98
Normal	TMA2 H1 cc2	47572250	209.74	Tumour	17538947	77.33
Normal	TMA2 H6 cc3	58639432	258.53	Tumour	828398	3.65
Normal	TMA2 H8 cc3	6594866	29.08	Tumour	654432	2.89
Normal	TMA2 I3 cc3	72469888	319.51	Tumour	16362593	72.14
Normal	TMA2 I8 cc	18678352	82.35	Tumour	18387654	81.07
Normal	TMA2 J7 cc3	2589038	11.41	Tumour	1.09E+06	4.81
Normal	TMA2 J8 cc3	26139685	115.25	Tumour	1583285	6.98
**Total**		**22681583.02**	**100.00**		**2953128.737**	**13.02**

Tumor intensity)-Normal intensity (%)	Tumor intensity -Normal intensity raw data	
-90.61	-20552234	Red font indicates a decreased value in tumor s
0.00	0	
-99.13	-22483249	
-61.44	-13936616	
-30.30	-6873208	
-40.98	-9294078	
-174.22	-39515592	
-54.15	-12281107	
-66.06	-14984414	
-106.71	-24203914	
-106.40	-24132136	
-33.01	-7486993	
-72.37	-16413642	
-64.36	-14597231	
-41.29	-9364296	
15.93	3612772	
-162.67	-36896556	
-178.88	-40572302	
-97.88	-22200833	
-113.06	-25644455	
-93.84	-21284982	
-236.39	-53617946	
-72.08	-16347809	
-68.38	-15508935	
-122.14	-27702580	
-118.07	-26779582	
-56.73	-12866608	
-154.08	-34946837	
-9.65	-2189537	
-115.42	-26179436	
-22.96	-5207093	
-33.83	-7672740	
3.56	807635	
-65.92	-14951670	
-47.82	-10847362	
-83.04	-18834315	
-7.68	-1742505	
-126.67	-28730470	
-155.05	-35168042	
-69.34	-15727991	
-55.60	-12611901	
-14.14	-3208182	
-280.98	-63731540	
-5.17	-1173524	
-26.09	-5918758	
-158.14	-35869471	

-6.01	-1362115
-185.33	-42034885
-201.12	-45616422
-15.17	-3440043
-132.41	-30033303
-254.88	-57811034
-26.19	-5940434
-247.37	-56107295
-1.28	-290698
-6.60	-1497000
-108.27	-24556400
	**-19728454.28**

samples when compared with the matched control value
