# Supplementary material for: Expression of protease activated receptor-2 is reduced in renal cell carcinoma biopsies and cell lines
Source: PLoS One. 2021 Mar 25;16(3):e0248983. doi: 10.1371/journal.pone.0248983 (PMC7993771; doi:10.1371/journal.pone.0248983)
Supplement: S2 File — (PDF) [file pone.0248983.s002.pdf]

ID	Normal	Matched Tumor area (Clear cell)
**3**	0.683020128	0.802922882
**4**	0.295248165	0.40332088
**5**	0.447512535	0.421420772
**6**	0.361817309	0.752623374
**7**	0.477420802	0.570381858
**10**	0.892959511	0.764894847
**16**	1.101905116	0.174342958
**18**	0.467595624	0.646176415
**20**	0.808507652	0.357661483
**21**	0.443395695	0.212175993
**26**	0.852634892	0.138376098
**27**	0.608501757	0.469761375
**35**	0.491978327	0.073302184
**42**	0.216134308	0.337587487
**44**	0.297989486	0.175961698
**51**	0.013368672	0.160985204
**56**	0.41754396	0.432268616
**58**	1.031683179	0.503477775
**59**	0.52304247	0.097395572
**61**	0.44288376	0.333324669
**69**	0.888842681	0.10732068
**73**	0.498270131	0.498270131
**80**	0.537747195	0.047366143
**83**	1	0.066754088
**90**	0.376311687	0.114625505
**105**	0.936272247	1.846764621
**106**	2.173469725	1.01395948
**113**	0.192109398	0.163799175
**119**	1.717130873	0.121581868
**Average**	**0.661906803**	**0.407200132**
